# What is the nocebo effect and does it apply to dentistry?—A narrative review

**DOI:** 10.1111/joor.13306

**Published:** 2022-03-09

**Authors:** Takeshi Watanabe, Mette Sieg, Sigrid Juhl Lunde, Pankaj Taneja, Lene Baad‐Hansen, Maria Pigg, Lene Vase

**Affiliations:** ^1^ Department of Psychology and Behavioural Sciences School of Business and Social Sciences Aarhus University Aarhus Denmark; ^2^ Department of Preventive Medicine Tokushima University Graduate School of Biomedical Sciences Tokushima Japan; ^3^ Section of Oral and Maxillofacial Surgery and Oral Pathology Department of Dentistry and Oral Health Aarhus University Aarhus Denmark; ^4^ Section of Orofacial Pain and Jaw Function Department of Dentistry and Oral Health Aarhus University Aarhus Denmark; ^5^ Scandinavian Center of Orofacial Neurosciences (SCON) Aarhus Denmark; ^6^ Scandinavian Center of Orofacial Neurosciences (SCON) Malmö Sweden; ^7^ Department of Endodontics Faculty of Odontology Malmö University Malmö Sweden

**Keywords:** dentistry, long term adverse effects, nocebo effect, oral surgery, pain, professional‐patient relations

## Abstract

**Background:**

Evidence for the nocebo effect, a phenomenon characterised by suboptimal treatment efficacy, worsening of symptoms, or the occurrence of adverse events caused by an individual’s negative treatment expectations, is growing across a multitude of medical fields. However, little attention has been paid to patients’ negative expectations and the nocebo effect within dentistry.

**Aim:**

This review summarises essential evidence of the nocebo phenomenon especially in relation to pain and drug administration. Subsequently, an overview of the current evidence of the nocebo phenomenon in the dental field is presented.

**Methods:**

A PubMed search was performed using keywords related to “nocebo,” “placebo,” “expectations,” and “dentistry.” In addition to the articles selected from the search, placebo/nocebo researchers and dental researchers added important references from their respective fields.

**Results:**

Although research on the nocebo effect in dentistry is limited, available current evidence suggests that the factors, which is related to the nocebo effect are likely to play a role in dental practice.

**Conclusion:**

Preliminary evidence from the review warrants further investigation into the nocebo effect in dentistry. Finally, based on the general knowledge of the nocebo effect, the review indicates fruitful arrays of research into the nocebo effect in dentistry.

## 1 INTRODUCTION

A patient's negative expectations about a treatment may lead to the experience of suboptimal treatment efficacy, worsening of symptoms or adverse events.^1^ This phenomenon, conceptualised as the nocebo effect, has been demonstrated in a multitude of experimental and clinical settings, suggesting that the implications of negative patient expectations are vast and should not be ignored.^2^, ^3^, ^4^ Little attention has been paid to the potential effect of negative patient expectations in dentistry, although the role of related concepts such as dental fear and anxiety are well‐documented.^5^ While many psychological factors may be associated with poor treatment outcomes, such as fear, anxiety, depression, disgust and poor coping,^6^, ^7^ the nocebo effect appears to make its distinct contribution specifically through negative expectations in the treatment situation and is the main focus of this review. Only within the last 5 years have a limited number of articles emerged, speculating the role of nocebo effects in dentistry.^8^, ^9^, ^10^, ^11^ Based on general knowledge of the nocebo phenomenon and current evidence from the field of dentistry, this review aimed to discuss the importance of extending our knowledge on the nocebo effect in dentistry. First, key nocebo terms are defined, followed by a brief overview of research on the nocebo effect(s). Then, evidence of the potential role of the nocebo effect in the context of dentistry is presented with suggestions for further research.

## 2 MATERIAL AND METHODS

A PubMed search was performed using keywords related to”nocebo,” “placebo,” “expectations” and “dentistry.” TW reviewed the search results and selected relevant references. In addition, placebo/nocebo researchers (MS, SJL, LV) and dental researchers (PT, LBH, MP) checked the search and added important references from their respective fields. As the nocebo field is still developing, and there is a certain heterogeneity in terms used within the field,^12^, ^13^ this article is a narrative review intended to provide an overview of the topic in relation to dentistry and identify gaps for future research.

## 3 WHAT IS THE NOCEBO EFFECT?

**FIGURE 1:**
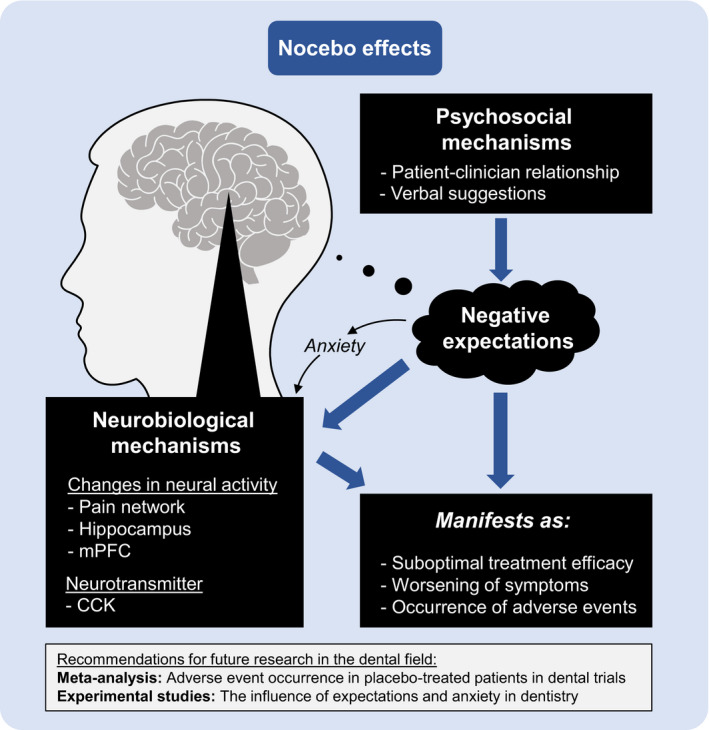
Important factors contributing to the nocebo effect and future areas for research into the nocebo effect in dentistry. Abbreviations: CCK, cholecystokinin; mPFC, medial prefrontal cortex

The term “nocebo” was originally introduced as the antithesis to the term “placebo”.^14^ Whereas the placebo effect refers to an improvement in symptoms caused by psychosocial factors such as positive expectations,^4^ the nocebo effect manifests as clinical worsening, suboptimal treatment efficacy or the occurrence of adverse events, presumably caused by negative expectations.^15^ Per definition, nocebo and placebo effects are non‐specific, which means that the perceived effect is not caused by any pharmacological agent, but rather by non‐specific common factors (eg expectations). It is likely that there are several different nocebo effects, just as there are several different placebo effects.^16^ One important mechanism of the nocebo effect appears to be the patient's negative expectations in relation to a treatment, which are connected to the patient‐clinician relationship, anxiety and verbal suggestions provided by the clinician.^1^ Figure 1 provides an overview of current evidence from the nocebo field.

Particularly negative verbal suggestions provided by the clinician, for example communicating a poor prognosis or disclosing potential side effects of a treatment, are associated with an ethical dilemma; sometimes called the ethical dilemma of the nocebo effect of informed consent.^17^ Patients have the right to, and also want to, be well‐informed about potential negative implications of a treatment (principle of autonomy).^18^ At the same time, clinicians should take care not to cause unnecessary harm (principle of non‐maleficence).^19^ Then, disclosing information about potential side effects violates the principle of non‐maleficence as it increases risk of side effect occurrence, while withholding this information violates the principle of autonomy. There is still no consensus on how to balance these two principles, yet this ethical dilemma highlights the importance of continuing to further our knowledge about the nocebo phenomenon.

As in clinical practice, nocebo in research is also complicated by ethics.^20^ Because manipulating expectations to induce negative outcomes can be ethically questionable, much of our knowledge about the nocebo phenomenon comes indirectly from investigating the proportion of adverse events in the placebo arms of randomised controlled trials (RCTs). In such trials, patients in both active treatment groups and placebo groups are informed about the potential adverse effects of the active treatment during the consent process. The underlying assumption is that any adverse events experienced by placebo‐treated patients must be caused by non‐specific factors such as the nocebo effect (ie expectations about adverse events causing adverse events).

Adverse events in patients receiving placebo has been observed in a multitude of diseases, such as various pain disorders, depression and cardiovascular disease.^3^ Around 50% of placebo‐treated patients experience adverse events,^3^ which suggests that a proportion of the adverse events experienced by patients receiving active treatments may similarly be caused by something other than the treatment itself. A systematic review of adverse events across various treatments and diseases suggested that only as little as 22% of the adverse events in the active treatment arm were caused by the treatment itself, and that the remaining 78% were caused by non‐specific factors.^21^ These non‐specific factors may be psychosocial factors contributing to the nocebo effect, but they may also be unrelated to the treatment situation, such as the natural history of the disease and regression towards the mean.^22^ Without the inclusion of no‐treatment groups to control for natural history and regression to the mean, real estimations of the “true” nocebo effect cannot be made. Nevertheless this evidence hints that negative expectations may contribute to the risk of adverse event occurrence across a multitude of different treatments and diseases.

Experimental studies, directly investigating the influence of negative verbal suggestion on adverse event occurrence, as well as symptom worsening and treatment efficacy, support indications from placebo‐controlled trials.^2^, ^23^ A systematic review found that providing information about the potential adverse events associated with a number of drugs significantly and consistently increased the risk of adverse event occurrence compared to omitting the information.^24^

As an example of symptom worsening, it has been demonstrated that verbally suggesting that an inert cream would worsen an allergic reaction resulted in a significant increase in allergic symptoms when compared to subjects informed that the cream would reduce the symptoms.^25^ Similarly, when patients were given intravenous morphine for post‐operative pain after thoracotomy, negative expectations increased pain ratings in patients who had their morphine delivery openly interrupted (ie a clinician explaining that pain treatment was stopped) compared to patients whose morphine delivery interruption was hidden (ie patients did not know that pain treatment had stopped).^26^

Negative verbal suggestion can also negatively influence treatment efficacy and even completely block the effect of active treatments.^2^ For example, the efficacy of remifentanil, a potent opioid analgesic, was examined for experimentally induced thermal pain on the right mid‐calf in healthy subjects, along with different types of verbal suggestions.^2^ While positive suggestion increased the efficacy of the drug, negative suggestion (suggesting that this drug would increase pain) resulted in a complete block of the analgesic effect. Interestingly, verbal suggestions not only influenced the patients' self‐reported pain ratings. Functional Magnetic Resonance Imaging showed a corresponding increased activity in the hippocampus, medial prefrontal cortex and the cerebellum as well as in core areas of the pain network (such as primary somatosensory cortex, midcingulate cortex, insula and thalamus), suggesting complex mechanisms for the attenuation of the analgesic effect.^2^ In line with this, a recent literature review suggests that nocebo effects and negative verbal suggestions are associated with changes in activity in cortical areas such as prefrontal, cingulate, insular and orbital cortices and subcortical areas such as the brainstem and amygdala.^27^ Furthermore, some evidence suggests that the cholecystokinin (CCK) antagonist, proglumide, which has been shown to enhance placebo effects,^28^, ^29^ blocks the nocebo effect in pain.^30^ This suggests that the CCKergic system is associated with nocebo hyperalgesia,^30^ which is further supported by the finding that CCK works as an opioid antagonist.^31^, ^32^, ^33^ Thus, although previously assumed to be associated with response bias, a growing body of evidence supports a psychoneurobiological basis of the nocebo effect.

The relationship between the patient and the clinician providing verbal suggestions has also been demonstrated to affect clinical outcomes.^34^ Although most research has focused on the positive effects of cultivating a good patient‐clinician relationship, some evidence suggests that a poor patient‐clinician relationship, resulting from a patient‐perceived lack of understanding and acceptance from the clinician, not only eliminates potential positive effects, but actually induces nocebo effects.^34^

Furthermore, a systematic review shows that anxiety, common in treatment situations and in itself linked to poor clinical outcomes,^35^, ^36^ is associated with nocebo effects.^37^ Although there is no consensus on the exact nature of this relationship, it is likely to be a bidirectional one. Anxiety may act as a moderator, such that being in a state of anxiety increases one's risk of experiencing nocebo effects.^37^ Furthermore, anxiety may mediate the effect of expectations on pain experience, such that expectations triggers anxiety, which in turn, activates brain areas facilitating pain experience.^38^, ^39^, ^40^

As in most other clinical settings, the patient‐clinician relationship, anxiety and verbal suggestion provided by the clinician during the consent process and throughout the course of treatment are non‐specific contextual factors that are also present in relation to dental treatments, making the presence of nocebo effects in dentistry likely. At present, however, only few letters and commentaries mention the possible existence of nocebo in dental treatment, urging dentists to recognise that negative verbal suggestion and non‐verbal communication during consultation may exacerbate patients' pain sensations.^8^, ^9^, ^10^, ^11^ The following section reviews current evidence of nocebo effects in dentistry.

## 4 DOES THE NOCEBO EFFECT APPLY TO DENTISTRY?

While no systematic reviews have investigated the pooled rate of adverse events in placebo arms of RCTs in dentistry, single studies indirectly suggest similar effects of non‐specific factors, such as expectations, within this field.^41^, ^42^, ^43^, ^44^ For example, clinical trials investigating the efficacy and safety of hydrogen peroxide in tooth whitening show that placebo‐treated patients experience adverse events such as hypersensitivity and gingival irritation,^41^, ^42^, ^43^ and in clinical trials of analgesics for pain following third molar (M3) removal, placebo‐treated patients experience drug‐specific adverse events such as nausea, headache and dizziness,^44^ hinting to the existence of nocebo effects in dental clinical trials.

Although the nocebo effect has yet to be directly investigated in relation to dentistry, a few experimental placebo studies provide some support for its existence.^45^, ^46^ In a randomised double‐blinded study, patients were administered either naloxone, which might increase patients' pain, or placebo following M3 extraction.^45^ Of the placebo‐treated patients, 61% reported increased pain levels similar to those receiving naloxone. Although the study did not allow for control of natural history of post‐surgical pain, these findings suggest that knowledge about a potential increase in pain due to the administered drug might induce a nocebo effect in a large proportion of patients in a dental clinical setting.^45^

The effect of the clinicians' expectations on patients' pain ratings following M3 removal has also been investigated.^46^ Patients were randomised into two groups: In the first group, clinicians, who administered the drug, believed that the patients could either receive intravenous placebo or naloxone, and in the second group, clinicians believed that patients could receive either intravenous placebo, naloxone or fentanyl (ie only in the second group did the clinicians believe that patients had a chance of experiencing pain relief). In both groups, all patients actually received a placebo. Patient pain ratings in the first group were significantly higher compared to the last group. This suggests that clinicians' own expectations about the efficacy of a treatment may influence patients' treatment outcomes.^46^

Moreover, although not investigated in relation to the nocebo effect, a few prospective studies suggest that patients' expectations are associated with the severity of pain during and after dental treatments. A study with patients needing emergency dental treatment showed that expected pain was associated with actual pain experience during treatment, and both were affected by negative emotional state.^47^ Similarly, a study showed that patients' expectations about the outcome of an upcoming endodontic treatment was associated with the development of persistent post‐treatment pain, regardless of case complexity. Patients expecting a “very good” treatment outcome were significantly less likely to experience persistent pain 6 months later compared to patients expecting merely a “fair to good” outcome.^48^

Adding to these considerations, several studies have investigated the influence of anxiety on dental outcomes.^49^, ^50^, ^51^ Evidence suggests that patients who present with strong dental anxiety tend to experience and recall higher levels of pain following tooth extraction.^52^, ^53^ Furthermore, anxiety and pain have been found to be associated in the setting of dental implant insertion.^51^ Considering the potential role of anxiety in the nocebo effect, the influence of anxiety in dentistry lends further support to the existence of nocebo effects in dentistry.

## 5 IMPLICATIONS AND CONCLUSION

Although research on the nocebo effect in the dental field is limited, the available current evidence demonstrates that verbal suggestions,^45^ patient‐clinician relationships,^46^ patients' expectations^48^, ^49^ and anxiety^5^ are factors likely to play a role in dental practice, just as it does in other clinical settings.^38^

To put this into a dental context, consider the following example: *It is Friday morning and a patient is sitting in the dental chair*, *about to undergo third molar extraction*. *Due to previous negative experiences of tooth extractions*, *he is very anxious about the procedure*. *The patient recently moved to the area and is meeting his new dentist for the first time*. *The dentist tells him about the procedure*, *and based on experience with previous patients informs him that he should expect postoperative discomfort and pain to a varying degree*. *The dentist advises the patient to make sure he has an adequate supply of painkillers available at home*, *and not plan too many activities for the weekend*. This example highlights the presence of non‐specific, context‐related factors (anxiety, patient‐clinician relationship, verbal suggestions and expectations) in dental practice. Further investigations into the potential negative effects of these non‐specific factors within dentistry is important, as we know that these factors contribute to nocebo effects in other clinical fields. The general nocebo literature shows that nocebo effects may be mitigated by withholding risk information^24^ or reframing this information in a positive way,^54^ by educating patients about the nocebo effect^55^ and by emphasising a strong patient‐clinician relationship.^56^ Thus, learning more about these nocebo effects may potentially open up for ways of optimising dental treatments.

Preliminary evidence from the current review warrants further investigations into nocebo effects in the dental field. A systematic investigation of adverse events in placebo‐treated patients from randomised dental trials, ideally including trials with a third no‐treatment group, could further illuminate the role of the nocebo effect in dentistry. Additionally, high quality experimental studies investigating the influence of negative expectations (eg through negative verbal suggestions) on both adverse event occurrence, symptom worsening and treatment efficacy in the dental setting, are needed. The dental field may also be a good area to further explore the relationship between anxiety and nocebo effects. Ultimately, this research will aid to clarify the role of the nocebo effect in dentistry, highlighting the potential implications for dental practice and providing hints of how the nocebo effect may be reduced.

## CONFLICT OF INTEREST

All authors declare no potential conflicts of interest with respect to the authorship and/or publication of this article.

## AUTHOR CONTRIBUTIONS

All authors contributed to conception, preparation of the manuscript and gave final approval and agree to be accountable for all aspects of the article. TW and MS drafted the first version of the manuscript. SJL, PT, LBH, MP and LV critically revised the manuscript.

### PEER REVIEW

The peer review history for this article is available at https://publons.com/publon/10.1111/joor.13306.

## Data Availability

Data sharing is not applicable to this article as no datasets were generated or analysed during the present study.
